# Diffuse left ventricular interstitial fibrosis is associated with sub-clinical myocardial dysfunction in Alström Syndrome: an observational study

**DOI:** 10.1186/s13023-015-0292-z

**Published:** 2015-06-24

**Authors:** Nicola C. Edwards, William E. Moody, Mengshi Yuan, Adrian T. Warfield, Robert Cramb, Richard B. Paisey, Tarekegn Geberhiwot, Richard P. Steeds

**Affiliations:** School of Clinical and Experimental Medicine, University of Birmingham, Birmingham, UK; Department of Cardiology, Queen Elizabeth Hospital, Birmingham, UK; Department of Pathology, Queen Elizabeth Hospital, Birmingham, England; Department of Biochemistry, Queen Elizabeth Hospital, Birmingham, UK; Department of Diabetes, Torbay Hospital, Torbay, UK; Department of Endocrinology, Queen Elizabeth Hospital, Birmingham, UK

**Keywords:** Alström syndrome, Myocardial fibrosis, Cardiac MRI, T1 mapping, Left ventricular function

## Abstract

**Background:**

Alström syndrome is a rare inherited ciliopathy with progressive multisystem involvement. Dilated cardiomyopathy is common in infancy and recurs or presents de novo in adults with high rates of premature cardiovascular death. Although Alström syndrome is characterised by fibrosis in solid organs such as the liver, the pathogenesis of related cardiomyopathy are not clear. To date it is not known whether diffuse interstitial myocardial fibrosis is present before the onset of heart failure symptoms or changes in conventional parameters of left ventricular function.

**Methods:**

In this observational study, 26 patients with Alström syndrome (mean age 27 ± 9 years, 65 % male, 24 h ABPM 130 ± 14 / 77 ± 9 mmHg) without symptomatic cardiovascular disease were recruited from a single centre and compared to matched healthy controls. All subjects underwent cardiac MRI (1.5 T) to assess ventricular function, diffuse interstitial myocardial fibrosis by measurement of extracellular volume on T1-mapping (MOLLI) and coarse replacement fibrosis using standard late gadolinium enhancement imaging.

**Results:**

Global extracellular volume was increased in Alström syndrome with wider variation compared to controls (0.30 ± 0.05 vs. 0.25 ± 0.01, p < 0.05). Left ventricular long axis function and global longitudinal strain were impaired in Alström syndrome without change in ejection fraction, ventricular size or atrial stress (NT-proBNP) (p < 0.05). Global extracellular volume was associated with reduced peak systolic longitudinal strain (r = −0.73, p < 0.01) and strain rate (r = −0.57, p < 0.01), increased QTc interval (r = 0.49, p < 0.05) and serum triglycerides (r = 0.66, p < 0.01). Nine (35 %) patients had diffuse mid-wall late gadolinium enhancement in a non-coronary artery distribution.

**Conclusion:**

Diffuse interstitial myocardial fibrosis is common in Alström syndrome and is associated with impaired left ventricular systolic function. Serial studies are required to determine whether global extracellular volume may be an independent imaging biomarker of vulnerability to dilated cardiomyopathy and heart failure.

## Background

Alström syndrome (ALMS) is a rare autosomal recessive syndrome (OMIM 203800) characterised by childhood onset retinal cone rod dystrophy, neuronal hearing loss, obesity and severe insulin resistance presenting with exaggerated features of the metabolic syndrome [1–4]. Estimates of the prevalence range from 1:10,000 [5] to fewer than 1:1,000,000 [6]. About 800 individuals diagnosed with ALMS have been identified worldwide. Idiopathic infantile dilated cardiomyopathy (ICM) presents acutely in nearly half of all ALMS subjects within the first few weeks of life [7, 8]. In survivors, the pathological process then appears to be fully reversible, as there are no persisting clinical, electrocardiographic or echocardiographic evidence of ventricular dysfunction, nor is there evidence of coarse myocardial fibrosis [8]. Cardiomyopathy may then recur or develop *de novo* in up to 65 % of adolescents and adults with high rates of morbidity and mortality, at which stage coarse fibrosis is evident on post-mortem [9]. This has also been demonstrated as patchy late gadolinium enhancement (LGE) using cardiac magnetic resonance imaging (CMR) [10, 11]. Although ALMS is characterised by diffuse fibrosis in other solid organs, there are only limited data identifying whether diffuse interstitial fibrosis may be present in the myocardium before onset of dilated cardiomyopathy [10]. Diffuse interstitial fibrosis may be missed on conventional late gadolinium enhancement (LGE) imaging, which relies on comparison of signal intensities between normal and abnormal myocardium within the same image [12]. This limitation is overcome with newer CMR T1-mapping techniques which can quantify the size of the extracellular volume (ECV). The primary aims of this study were; 1) to establish whether diffuse interstitial myocardial fibrosis is present in asymptomatic adult subjects with ALMS, 2) if present, determine whether this is associated with change in left ventricular function.

The profound metabolic disturbances which characterise ALMS include severe insulin resistance, activation of the renin angiotensin aldosterone system and raised serum triglycerides - each of which has a potential role in the development of myocardial fibrosis [2, 13–15]. Thus, although ALMS is rare, it offers a model of disease processes, including metabolic syndrome, that are of increasing importance to a much wider population. A secondary aim of this study was to identify associations between potential metabolic changes in ALMS that might mediate myocardial fibrosis.

## Methods

### Study design

A propsoective cohort observational study performed between March 2012 and November 2013.

### Patients & setting

In total, 26 consecutive adults with genetically proven ALMS attending the National Centre for Rare Disease at the Queen Elizabeth Hospital Birmingham, England were studied prospectively as part of standard clinical care. Two other patients were seen at the National Centre during this time period but did not proceed with CMR due to contra-indications (one permanent pacemaker; one with a cochlear implant). Patients were compared to gender and age matched healthy controls with no history of cardiac disease recruited as part of an on-going clinical study [16].

### Ethics statement

This study was approved by the National Research Ethics Service and all patients provided written informed consent to participate (12/WM/0250).

### Cardiac MRI

Consent was obtained for contrast enhanced CMR (1.5 T Avanto, Siemens Healthcare, Erlangen, Germany). Left ventricular (LV) dimensions, volumes and mass were acquired in line with standard CMR protocols [17]. Mitral annular plane systolic excursion (MAPSE) were measured on standard 4-chamber SSFP cine images, using the straight-line distance travelled by the lateral mitral annulus from end-diastole to end-systole. CMR-tagging (SPAMM) was performed in the horizontal long-axis (4 chamber) image using prospective electrocardiographic gating [18]. Late enhancement (LGE) imaging was performed 7–10 min after 0.15 mmol/Kg of gadolinium contrast bolus (Gadovist Bayer Health Care). LGE is an imaging technique that characterizes the myocardium by detecting patterns of disease including reparative fibrosis and infarction. Differences in the “wash out” kinetics of damaged myocardium creates relative differences in signal intensity within the myocardium to highlight areas that are abnormal (white) compared to areas that are normal (black). These white areas correspond to macroscopic reparative fibrosis. The technique is reliant on the heart containing areas of normal myocardium to reveal the difference in signal intensity – which is one reason why the technique may fail to detect diffuse myocardial disease throughout the ventricle. T1 mapping is a newer technique which has the advantage that there is no need for “normal” myocardium against which to compare T1 values within the same heart and is thus capable of detecting diffuse fibrosis, as has been shown in other conditions [9] and is postulated in ALMS. Therefore, in addition to standard LGE, short-axis images at LV basal and mid-ventricular levels were acquired using an ECG-gated modified Look-Locker inversion recovery sequence (MOLLI) sequence for T1 mapping before contrast and at 15–20 min after bolus contrast administration [19]. No patient had complications arising from the use of Gadolinium contrast.

### CMR image analysis

Analysis was performed off-line using Argus Software (Siemens®) according to the Society for Cardiovascular MR guidelines for reporting CMR with parameters indexed to height and body surface area where appropriate [20]. Tagged images were analyzed with CIMTag2D software (Cardiac Image Modelling, University of Auckland, Auckland) to calculate global peak systolic strain, peak systolic strain rate (myocardial deformation) and peak early diastolic strain rate (an indicator of diastolic function) [18].

Quantitative parametric images of myocardial ECV were generated with manual contouring to define a region of interest (ROI) within the LV myocardium in the ventricular septum (4-chamber view) and at basal and mid ventricular level (short-axis) on matched pre and post contrast images (Siemens, Erlangen, Germany). Care was taken to avoid contaminating T1 measurements at the myocardial-blood pool interface. T1 time in each ROI was recorded and used to calculate myocardial extracellular volume (ECV) by validated formulae [21].

$$ \mathrm{E}\mathrm{C}\mathrm{V} = \left(\left(\Delta \mathrm{R}1\ \mathrm{myocardium}\right)/\left(\Delta \mathrm{R}1\ \mathrm{blood}\ \mathrm{pool}\right)\right)\left(1\hbox{-} \mathrm{H}\mathrm{c}\mathrm{t}\right) $$

HCT refers to the haematocrit recorded on a venous blood sample at the time of scan, ΔR1 = 1/T1 post contrast – 1/T1 pre contrast. The ECV was assessed in the left ventricle from the basal and mid ventricular levels, averaged to yield a “global ECV” measurement. Further details on the methods used have been published [21, 22].

### Biochemistry

Venous blood samples were collected for routine haematology, biochemistry and lipid profiles. Serum N-terminal pro B Natriuretic peptide (NT-proBNP, ng/L) was measured by sandwich immunoassay with magnetic particle separation and chemiluminescent detection on an E170 analyser (Roche Diagnostics, Burgess Hill) with a lower limit of detection of 0.6 pmol/L. Plasma renin was measured using an immunoradiometric assay (Cis Bio, IBA Molecular UK, Guildford) with age and position reference intervals and a detection limit of 5 ng/L. Aldosterone was measured by competitive radioimmunoassay (Coat-a-Count, Siemens Medical Solutions Diagnositics, Llanberis.) which has reference intervals dependent on position with a detection limit of 60 pmol/L. C-peptide (pmol/L)/insulin (pmol/L) were also measured (Royal Surrey County Hospital, Guildford).

### Blood pressure

Subjects underwent 24 h ambulatory blood pressure monitoring using a British Society of Hypertension approved protocol [23].

### Electrocardiogram

Standard 12-lead electrocardiograms were recorded in each subject within 24 h of the CMR scan, with measurement of PR interval, QRS duration and QTc intervals using standard methodology (Bazett’s formula for QTc).

### Statistical analysis

Continuous variables are expressed as mean ± standard deviation (SD) if normally distributed or median (interquartile range) if non-normally distributed by the Shapiro-Wilk test. Paired group comparisons were performed using independent T-test, Mann–Whitney and one-way analysis if variance with Bonferroni post hoc tests, as appropriate. Correlations between variables were assessed by Pearson if normally distributed and Spearman analyses if non-normally distributed. A multivariable regression model was used to assess predictors of ECV using variables associated with myocardial fibrosis. Statistical tests were two tailed and a p-value <0.05 was considered to indicate statistical significance.

## Results

In total, 26 ALMS patients were compared with healthy controls (mean age 27 ± 9 vs. 27 ± 6 years, p = 0.24). Subject genetic and demographic profiles are presented in Table 1 and Table 2 respectively. A history of infantile cardiomyopathy was documented in eight ALMS patients. One patient had end-stage renal disease (ESRD) requiring dialysis. One patient had a history of prior myocardial infarction. All patients were NYHA class I.

**Table 1:** Individual patient demographic profile

Patient	Age	Gender	Ethnicity	BMI	ALMS	Protein	ALMS	Protein	Infantile CM
					mutation	change	mutation	change	
1	21	Male	White British	32.6	c.6526C > T	p.Gln2176X	c.11101C > T	p.Arg3701X	Yes
2	19	Male	White British	27.1	c.6526C > T	p.Gln2176X	c.11101C > T	p.Arg3701X	No
3	18	Male	White British	32.3	c.8932C > T	p.GLN2978X	c.5356A > G	p.ASN1786Asp	Yes
4	17	Male	White British	26.4	c.10769delC	p.Thr3590LysfsX6	c.5356A > G	p.Asn1786Asp	Yes
5	20	Male	White British	31.5	c6526C > T	p.Gln217X	c11101C > T	pArg3701X	No
6	49	Male	White British	31.9	c.10483C > T	p.Gln3495X	c.10775delC	P.Threo3592LysfsX6	No
7	40	Male	White British	38.4	c.1729delA	p.Arg577Glyfsx17	c.10477C > T	p.Gln3493X	No
8	25	Female	Mixed race	30.0	c.2218dupA	p.ThreoAsnfsX2	c.1069C > T	p.Arg3657X	No
9	45	Male	White British	35.3	c.1874A > G	p.His623Arg			No
10#	44	Male	White British	28.2	c.10769delC	p.Thr3590LysfsX6	c.11410C > T	p.Arg38404X	Yes
11	21	Female	White British	26.4	c6584delA	p.Lys2195SerfX10	C1008_1009delT	pCys336fsX1	Yes
12	22	Female	White British	27.6	c.6823C > T	p.Arg2275X	c.9535C > T	P.Arg3179X	No
13	26	Male	White British	22.7	c.6823C > T	p.Arg2275X	c.9535C > T	P.Arg3179X	No
14	29	Male	White British	31.6	c.6895delG	p.val2299TrpsX43	c.11443C > T	p.Gln3815X	No
15	43	Male	White British	35.2	c.8995C > T	p.Gln3493X	c>9001C > T	Gln3001	No
16	29	Female	White British	27.0	c.8002C > T	p.arg2668X	c.10879C > T	pArg3627x	No
17	18	Male	BritishPakistani	27.8	c.4937C > A	p.Ser1646X			No
18	20	Male	BritishPakistani	36.6	c.7544-200_767 + 1110del				No
19	19	Male	BritishPakistani	26.8	c.7544-200_767 + 1110del				Yes
20	21	Female	BritishPakistani	29.6	c.5075delC	p.Pro1692LeufsX39	c.5075delC	p.Pro1692LeufsX39	Yes
21	24	Female	White British	26.5	Result awaited*	-	-	-	No
22	20	Male	BritishPakistani	25.9	c.4937C > A	p.Ser2646STOP			Yes
23	19	Female	British Indian	19.4	c.2041C > T	p.Arg681X	c.2041C > T	p.Arg681X	Yes
24	23	Male	British Indian	28.9	c.2041C > T	p.Arg681X	c.2041C > T	p.Arg681X	No
25	32	Female	White British	25.7	C11101C > T	p.R3701X			No
26	20	Female	White Australian	37.7	c.7911dupC	p.Asn2638Glnfs24			No

#End-stage kidney disease on dialysis

*Genetic data pending at time of publication. Patient fulfills the clinical diagnostic criteria for ALMS on serial assessment

**Table 2:** Demographic, haemodynamic and biochemical data

	Controls	ALMS
Number *n*	26	26
Age years	27 ± 6	27 ± 9
Male *n* (%)	17 (65 %)	17 (65 %)
Height (m)	1.74 ± 0.09	1.61 ± 0.09**
Weight (Kg)	76 ± 16	77 ± 16
Body Mass Index	25 ± 4	30 ± 5**
Haemoglobin (g/dL)	14.3 ± 1.2	14.0 ± 2.2
eGFR (ml/min/1.73 m^2^)	87 ± 8	73 ± 25*
Renin (pg/ml) ^a^	-	41 (18–153)
Aldosterone (pg/ml) ^a^	-	97 (67–220)
NT-pro BNP (ng/L) ^a^	38 (4–75)	37 (17–89)
Cholesterol (mmol/L)	4.6 (0.8)	5.1 ± 1.3
Triglycerides (mmol/L) ^a^	1.0 (0.6–1.4)	2.5 (1.5–4.1)**
High Density Lipoprotein (mmol/L)	1.4 (0.3)	0.9 ± 0.3**
Glucose (mmol/L) ^a^	4.7 (4.2–5.0)	6.1 (4.6–15.0)*
HbA_1c_ (%)	-	7.9 ± 2.7
Insulin (pmol/L) ^a^	-	858 (239–1760)
C-peptide:Glucose ratio (ng/ml:mg/dl) ^a^	-	4.2 (2.2–6.2)
ACR (mg/mmol) ^a^	0.1 (0.05–0.2)	1.5 (0.25–34)
Urate (mmol/L)	286 ± 67	409 ± 111*
24 h daytime SBP (mmHg)	119 ± 9	130 ± 14*
24 h daytime DBP (mmHg)	72 ± 7	77 ± 9*

*ACR* albumin/creatinine ratio, *ALMS* Alström Syndrome, *DBP* diastolic blood pressure, *eGFR* Estimated glomerular filtration rate, *HBA*_*1c*_ glycated haemoglobin, *NT-pro BNP* N-terminal pro B natriuretic peptide, *SBP* systolic blood pressure

Value ± standard deviation, ^a^median (inter-quartile range), *p* = * < 0.05, *p* = ** <0.01

Patients with ALMS demonstrated marked metabolic derangements (Table 2). All were insulin resistant. Seventeen patients had diabetes and five patients had impaired glycaemia. Mean C-peptide: Glucose ratio (a marker of insulin resistance) was increased. Serum triglyceride levels were also elevated with reduced serum HDL levels compared to controls. NT-proBNP was increased above normal range (>200 ng/L) in only 2 patients. One of these patients had ESRD and the subject was below the threshold for diagnosis of heart failure (<400 ng/L).

### Cardiovascular structure, function & tissue characterization

#### Ventricular volumes and function

Patients with ALMS had reduced absolute and indexed LV volumes compared to controls but did not differ when indexed for height (to allow for obesity). Absolute LV mass did not differ between groups but was increased when indexed to height^2.7^. There was no difference in LV ejection fraction (EF) but longitudinal function (MAPSE) was reduced (Table 3).

**Table 3:** Cardiac MRI Functional and Fibrosis data

	Controls	ALMS		ALMS	
			ECV < ULN	ECV > ULN	ECV > ULN
				NO LGE	LGE
Number *n*	26	26	12	5	9
Mean global ECV	0.25 ± 0.01	0.30 ± 0.05*	0.25 ± 0.02	0.31 ± 0.03^a^	0.34 ± 0.05^b^
Global pre contrast T1 (ms)	970 ± 11	949 ± 35	934 ± 34	952 ± 33	972 ± 35
Global post contrast T1 (ms)	522 ± 58	465 ± 61*	472 ± 54	440 ± 47	437 ± 43
Diffuse LGE *n*	0	9	0	0	9
LVEDV (ml)	133 ± 29	105 ± 23**	107 ± 31	100 ± 13	108 ± 25
LVEDV index (ml/m^2^)	69 ± 12	57 ± 11**	57 ± 13	56 ± 11	58 ± 13
LVESV(ml)	43 ± 13	38 ± 15	37 ± 15	32 ± 8	43 ± 19
LVESV index (ml/m^2^)	22 ± 6	20 ± 8	20 ± 7	18 ± 5	23 ± 10
LVEF (%)	68 ± 5	66 ± 8	67 ± 7	69 ± 5	62 ± 11
LV mass (g)	115 ± 31	104 ± 27	106 ± 27	87 ± 13	112 ± 34
LV mass index (g/m^2^)	60 ± 14	56 ± 12	56 ± 10	48 ± 8	60 ± 16
LV mass/ Height^2.7^ (g/m^2.7^)	25 ± 6	29 ± 7*	28 ± 6	24 ± 6	32 ± 9
LA volume (ml/m^2^)	33 ± 8	35 ± 15	29 ± 8	40 ± 14	39 ± 15
MAPSE (mm)	17 ± 2	12 ± 2*	13 ± 2	13 ± 3	11 ± 3
Global long SS (%)	14.7 ± 2.3	11.2 ± 2.6**	12.9 ± 1.0	10.9 ± 2.0^a^	9.6 ± 1.9^b^
Global long SSR (s^−1^)	0.83 ± 0.15	0.57 ± 0.11**	0.63 ± 0.16	0.53 ± 0.11^a^	0.52. ± 0.15^b^
Global long ESR (s^−1^)	0.62 ± 0.15	0.49 ± 0.18*	0.54 ± 0.15	0.44 ± 0.19	0.41 ± 0.19

*EDV* end-diastolic volume, *ESV* end-systolic volume, *EF* ejection fraction, *LA* left atrium, *LGE* late gadolinium enhancement, long; longitudinal, *LV* left ventricle, *MAPSE* mitral annular plane systolic excursion, *RV* right ventricle, *TAPSE* tricuspid annular plane systolic excursion, *SS* systolic strain, *SR* systolic strain rate, *ESR* early diastolic strain rate, *ULN* ECV above the upper limit (0.279) observed in matched control

^a^ *p* < 0.05, Bonferroni post-hoc tests for differences ECV above upper limit of normal controls vs. increased ECV no LGE

^b^ *p* <0.05, Bonferroni post-hoc tests for differences ECV above upper limit of normal controls vs. increased ECV with LGE

ANOVA * *p* <0.05, ** <0.01, ALMS vs. controls

### Myocardial tagging

Left ventricular global longitudinal peak systolic strain and strain rate were reduced in ALMS (Table 3).

### T1 mapping

**Fig. 1:**
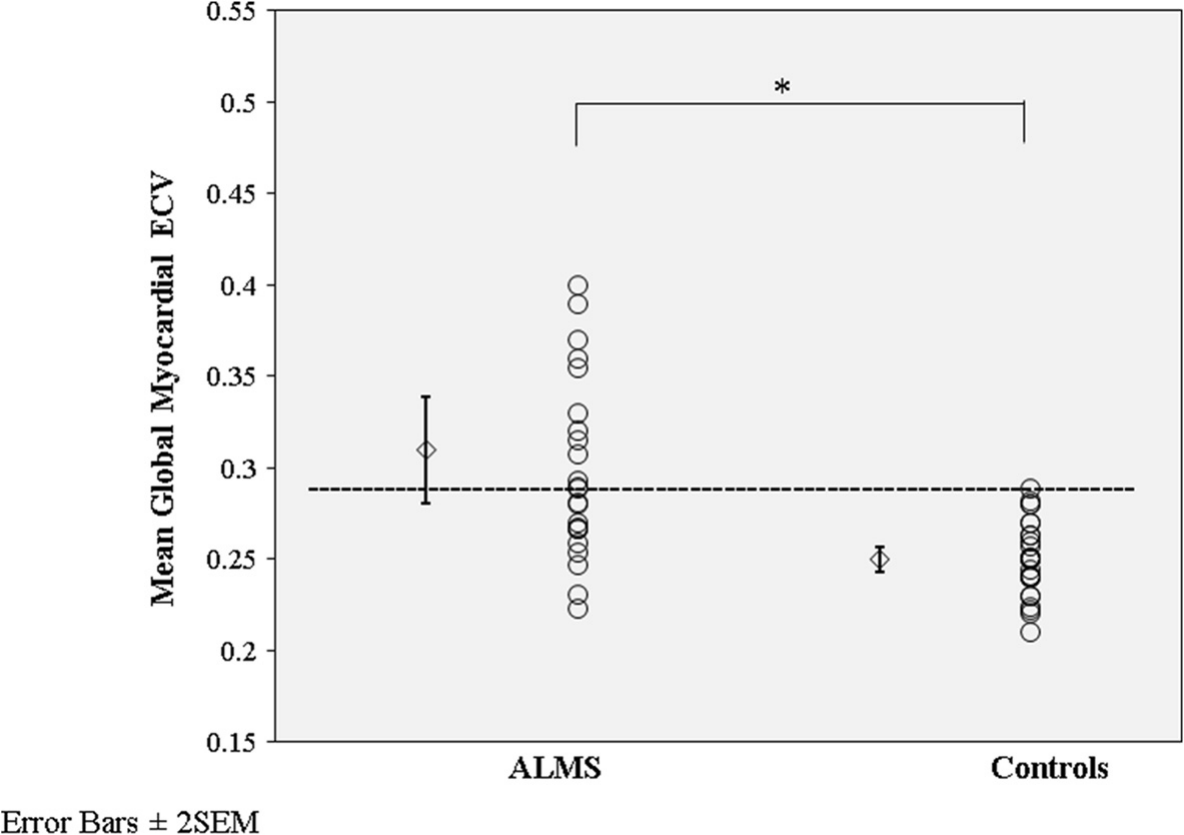
Box scatter plots of global extracellular volume (ECV) in patients with ALMS and controls. Error bars are standard error of the mean x2. Dashed horizontal line defines the upper value within the matched normal population (0.279). ECV was assessed in the left ventricle from the basal and mid ventricular levels and averaged to yield a “global ECV” measurement. * p <0.05

Mean global ECV was increased compared to controls (Table 3) but with wider variation and cross-over with the normal population at the lower end of the range (Fig. 1). Patients with ALMS had reduced LV longitudinal function (MAPSE 12 mm ±2 vs. 17 mm ± 2, p < 0.01), reduced global longitudinal strain (11.2 % ± 2.6 vs. 14.7 % ± 2.3, p < 0.01), strain rate (0.58 s^−1^ ± 0.11 vs. 0.83 s^−1^ ± 0.15, p < 0.01) and reduced early diastolic strain rate (0.49 ± 0.18 s^−1^ vs. 0.62 ± 0.15 s^−1^, p < 0.05). Fourteen patients (54 %) had an ECV above the upper limit (0.279) for controls. Five patients had ECV above the upper limit for matched normal controls in the absence of LGE (Table 3). There was no difference in mean pre-contrast myocardial T1 times between ALMS and controls (Table 3). Indeed, 11 ALMS patients (41 %) had T1 times within the range for matched controls (943 ms). Post contrast T1 times were reduced in ALMS (Table 3).

### Late gadolinium enhancement

**Fig. 2:**
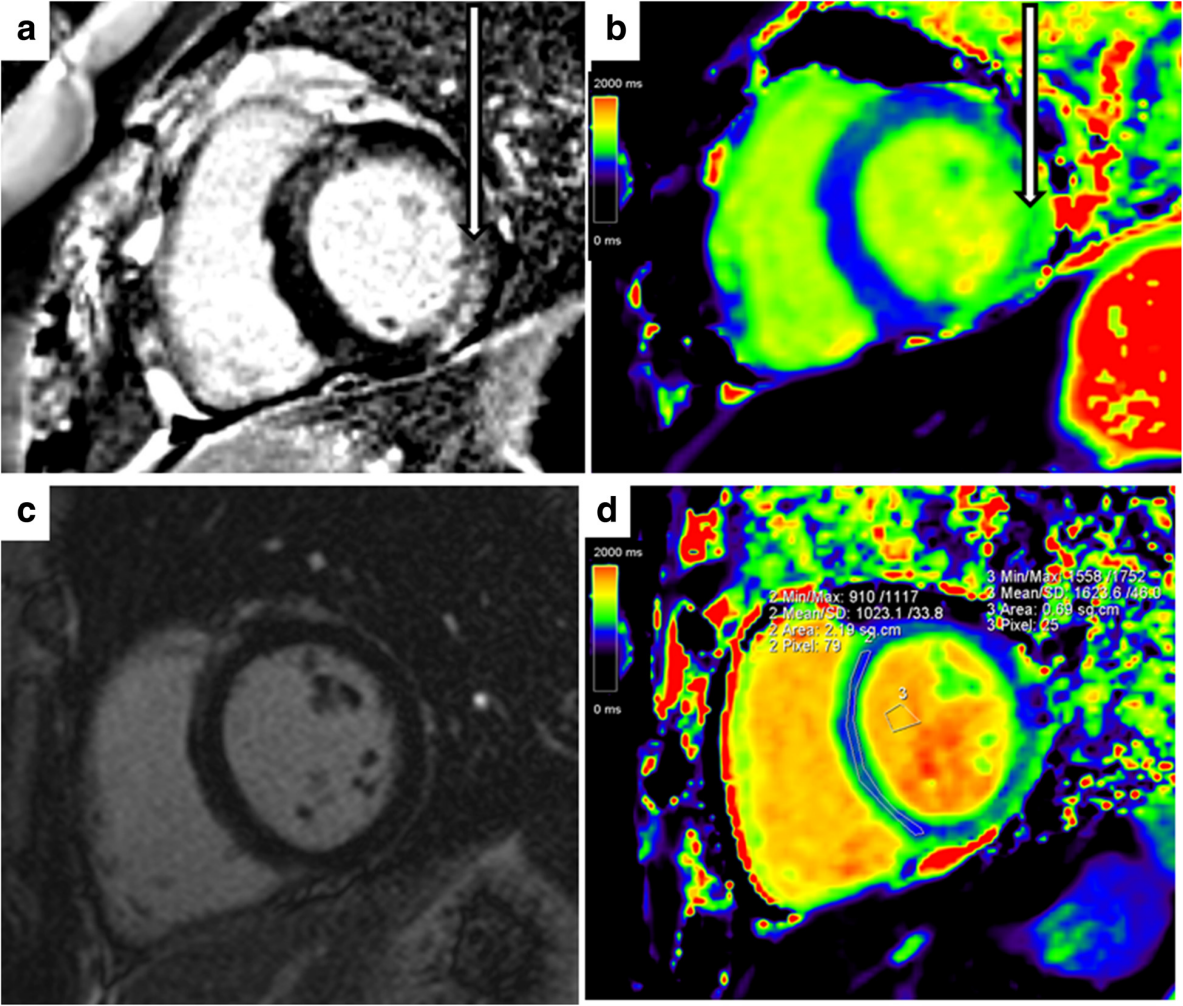
Examples of late gadolinium enhancement and corresponding pre-contrast colour T1 maps in patients with ALMS. **a** Clear late enhancement (arrow) in the basal infero-lateral wall. **b** Corresponding high T1 (1057 ms) on the T1-map (arrow). **c** No late enhancement in the left ventricular myocardium. **d** Corresponding areas with high T1 (1043 ms) on the T1-map in the septum and infero-lateral wall (green)

Late gadolinium enhancement in a diffuse, non-coronary artery pattern was present in 9 (35 %) patients with ALMS (two with a history of infantile cardiomyopathy). An example is shown in Fig. 2. Compared to ALMS subjects with no LGE but increased ECV, subjects with LGE did not have increased ECV (0.34 ± 0.05 vs. 0.31 ± 0.03, p = 0.22) and no differences in LV volumes, ejection fraction or deformation (Table 3).

### ECG

All ALMS patients were in sinus rhythm with normal atrial activation (PR interval 143 ms ± 16), ventricular depolarisation (QRS duration 88 ms ± 16) and ventricular repolarisation (QTc 416 ms ± 24 and 426 ms ± 24 for males and females respectively). Patients with ECV above the upper limit of the range for matched controls had increased QTc (430 ± 21 ms vs. 412 ± 18 ms, p < 0.05).

### Myocardial fibrosis

**Fig. 3:**
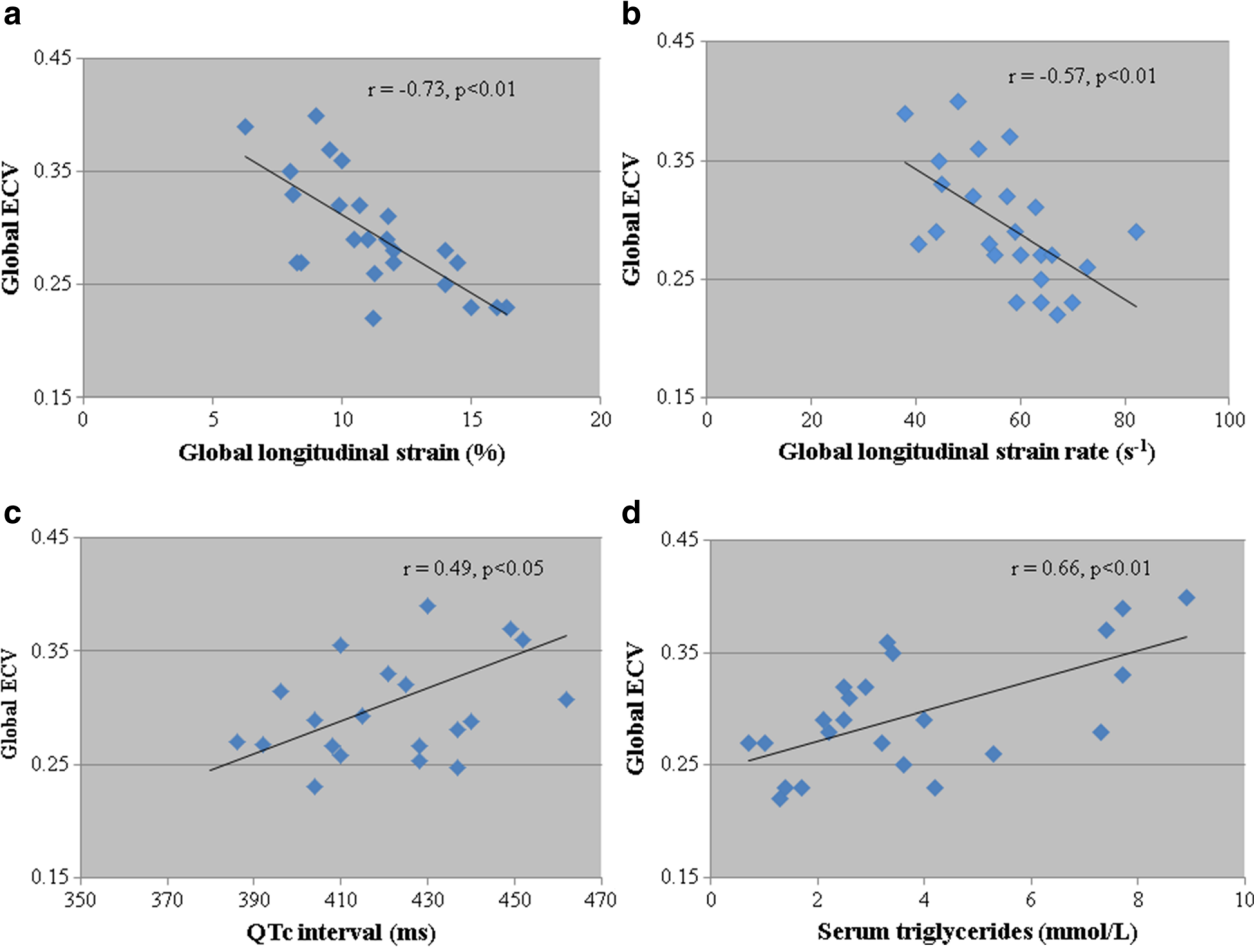
Association of diffuse myocardial fibrosis with markers of lipid metabolism, systolic function and cardiac electrical activity. Scatter plots demonstrating the association between global extracellular volume and **a**) global longitudinal strain, **b**) global systolic strain rate, **c**) 12 lead ECG QTc interval and **d**) serum triglycerides in patients with ALMS

ECV correlated with age (r = 0.50, p < 0.05), global longitudinal peak systolic strain (r = −0.73, p < 0.01), peak systolic strain rate (r = −0.57, p < 0.01), MAPSE (r = −0.39, p < 0.05), but not ejection fraction, NT-BNP or LV mass/height^2.7^. Serum triglycerides (r = 0.66, p < 0.01) and QTc (r = 0.49, p < 0.05) also correlated with ECV (Fig. 3). These factors were entered into a multivariable linear regression model with ECV as the dependent variable; r^2^ = 0.75, p < 0.01. Global longitudinal strain remained an independent predictor of ECV (unstandardized β coefficient −0.01, 95 % CI −0.02-0.004, p < 0.01).

### Patient events

**Fig. 4:**
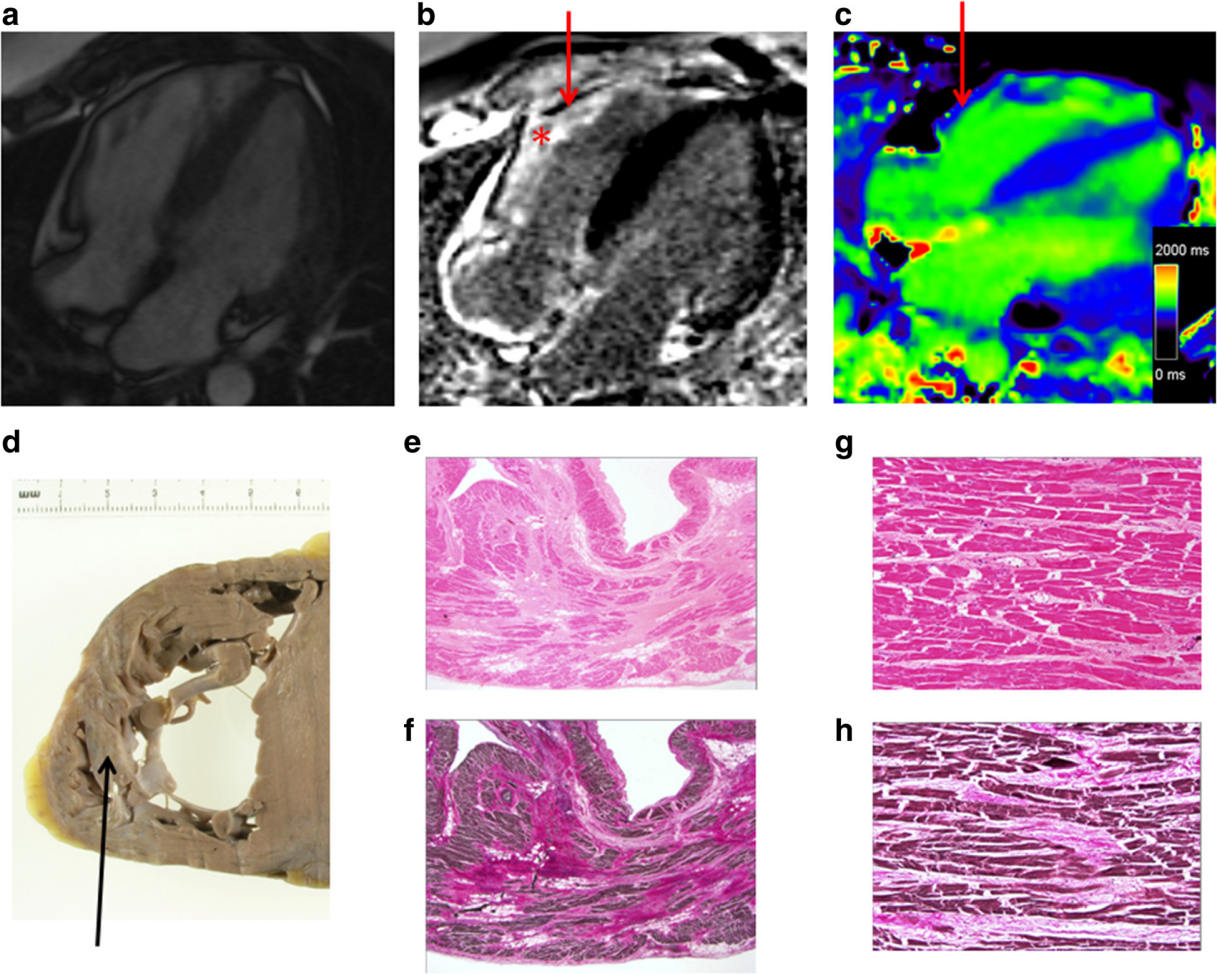
Myocardial fibrosis on CMR and post-mortem specimens from a patient with ALMS. **a-c**) 4 chamber CMR imaging; **a** cine, **b** late gadolinium enhancement and **c** T1 mapping. Note the extensive LGE in the RV (*) with corresponding low T1 on the post-contrast MOLLI. **d** Corresponding macroscopic image from autopsy of the RV with strands of sub-endocardial fibrosis seen as pallor within the RV (black arrow). **e-f** Corresponding low power views of part of the free wall of the right ventricle demonstrating patchy swathes of interstitial fibrosis replacing myocardiocytes with more subtle pericellular fibrous expansion. The fibrous tissue stains pink with H&E and red with EHVG (*H&E & EHVG, original magnification X1.25*). **g-h** Comparable intermediate magnification photomicrographs of the free wall of the right ventricle depicting more dispersed interstitial fibrosis enclaving groups of myocytes and focally insinuating between individual muscle cells. The fibrous tissue stains pink with H&E and red with EHVG (*H&E stain, original magnification X1.25*)

Two patients (Table 1: patient 7 aged 40 and patient 10 aged 44 years) died over the study period. Both deaths occurred unexpectedly following a short respiratory tract infection which led to cardiac decompensation and multi-organ failure in the intensive care setting. Neither patient had a history of infantile cardiomyopathy but both had a history of severe hypertriglyceridaemia in teenage years (>50 mmol/l) with pancreatitis and raised plasma renin (>3000 ng/L) when normotensive. Furthermore, both had extensive patchy LGE on CMR, elevated global ECV (0.33 and 0.41 respectively) and reduced myocardial deformation despite a normal LV ejection fraction. On autopsy of one patient, there was extensive fibrosis in a non-ischaemic pattern in the RV which corresponded with the extensive LGE on CMR (Fig. 4) and evidence of a chronically occluded right coronary artery with extensive collateralisation.

## Discussion

Interstitial myocardial fibrosis assessed using CMR T1 mapping, was evident in over 50 % of patients with ALMS. Coarse fibrosis within the LV myocardium was detected in keeping with previous reports but a further 20 % of patients were shown to have interstitial fibrosis missed using conventional LGE alone. Expansion of the ECV was associated with impaired global LV systolic deformation, impaired LV diastolic function and increased QT interval.. Impairment of global longitudinal strain and strain rate preceded any change in ejection fraction and without abnormality of conventional markers of elevated LV end-diastolic pressure and atrial stretch, including both NT pro-BNP and left atrial volume. This finding raises the possibility that surveillance of ALMS for evidence of cardiomyopathy using NT pro-BNP may be inadequate. In contrast to previous echocardiographic studies [24] that have reported ‘low-normal’ LV ejection fraction, use of CMR in young adults with ALMS indicates that patients have equivalent conventional values of ventricular size and EF to healthy controls but reduced myocardial deformation and diastolic function. These data support the concept that expansion of the ECV and reduced myocardial deformation may be more powerful predictors of adverse cardiovascular outcome than ejection fraction [22].

Although ALMS is a rare autosomal recessive genetic disorder, there is much interest in understanding the disease as a monogenic model for end-organ fibrosis which has been proven to affect the liver and kidneys [4]. The increase in diffuse interstitial myocardial fibrosis (as measured by ECV) and prolongation of the QTc interval on 12 lead electrocardiograms in this study are consistent with the limited autopsy data available in 5 ALMS patients in whom moderate to severe interstitial myocardial fibrosis was found [9]. The data are also consistent with a smaller study of 8 patients, only 3 of whom had normal LVEF at the time of CMR, in which T1 scout sequences were used to identify lower pre-contrast T1 relaxation as a measure of diffuse fibrosis [10]. Further studies using T1-mapping have demonstrated that diffuse interstitial fibrosis is prominent in diabetic patients [25], correlates with insulin resistance [26] and is associated with adverse prognosis [27]. In keeping with these data, our ALMS cohort displayed shorter myocardial post contrast T1 times and reduced deformation which is not unexpected given the high prevalence of impaired glycaemia. Furthermore, the correlation between expansion of the ECV and elevated triglycerides in our study may be consistent with poor diabetic control and supports the latter as a potential mediator. The mechanism behind the development of fibrosis within the myocardium is not known. It has recently been shown that ALMS1 is an important molecule for cell cycle regulation in perinatal cardiomyocytes and that deficiency of the protein may lead to a mitogenic cardiomyopathy [28]. This was supported by the histological diagnosis of mitogenic cardiomyopathy in two siblings from consanguineous parents of Turkish origin who died in infancy from dilated cardiomyopathy [29]. This may explain perinatal mortality but other mechanisms may be important in the development of adult cardiomyopathy, such as the onset of adiposopathy, wherein adiposite enlargement may be associated with inflammation and insulin resistance [30].

We acknowledge that while mean global ECV was higher than controls in this study, expansion of the ECV was not universal and there was considerable dispersion of ECV values within the range of normal healthy controls. Indeed, 40 % of patients had a native pre-contrast myocardial T1 values below the cut off for healthy volunteers (943 ms) raising the possibility of myocardial fat deposition or pseudonormalization of T1 due to the combined presence of lipid and fibrosis within the LV. This significant inter-individual variation suggests that development of fibrosis is not an inevitable consequence of the genetic defect alone and that environmental factors may also play an important role. Further serial studies are warranted in ALMS to determine whether those with higher ECV are susceptible to adverse cardiovascular outcomes, as has been shown to be the case in other populations [27].

Despite the fact that ALMS patients are characterised by obesity, severe insulin resistance and metabolic syndrome [13, 2], reduction in myocardial deformation was not due to myocardial infarction, since the distribution of LGE was neither subendocardial nor consistent with a coronary artery territory. It is possible that expansion of the ECV with increased diastolic stiffness in association with subclinical ventricular dysfunction [31] may have contributed to the failure of the two adult patients to survive intercurrent respiratory tract infections, as occurred in this series. It is also interesting to speculate whether expansion of the ECV is responsible for prolongation of the QTc and whether this predisposes individuals to lethal arrhythmia. It is noteworthy that despite extensive fibrosis in the two patients that died, both were asymptomatic before developing a chest infection.

An unusual feature of ALMS is the occurrence of infantile cardiomyopathy within the first few months of life which, if not fatal during the acute stage, may be followed by an apparent complete recovery [8]. Adults with ALMS have an increased risk of adverse cardiovascular outcomes but this risk does not appear to be heightened by a history of infantile cardiomyopathy. In our study, there was no relationship between exposure to infantile CM and the severity of diffuse interstitial fibrosis, presence of coarse scarring on LGE or reduction either in LVEF or in myocardial deformation. These data are consistent with the theory that the pathophysiology of early (infantile) and late cardiovascular morbidity and mortality is different. Coarse replacement fibrosis documented with LGE does not appear to be simply a consequence of scarring from severe cardiomyopathy in childhood, a concept supported by two previous smaller studies in ALMS [11, 14]. In addition, the recent work by Shenje et al. [28] and Louw et al. [29] have suggested a mitogenic cardiomyopathy may underly infantile dilated cardiomyopathy, although these changes have not been documented in adult post-mortem hearts. Inferences relating to infantile cardiomyopathy from our data however are limited by the fact that our study only included adult survivors, since patients under the age of 16 years are excluded from referral to the UK National Centre at Queen Elizabeth Hospital.

### Limitations

Given the small sample size and narrow age range of patients with this rare disease, we were underpowered to show some relationships reported previously in ECV [32]. This is a cross-sectional, observational study, and hence it is only possible to speculate on the aetiology of diffuse fibrosis and whether it is represents an intermediate phenotype in ALMS between cardiovascular health, coarse myocardial fibrosis and ultimately LV failure. The extent to which diffuse interstitial fibrosis is related to the primary effect of the *ALMS1* mutation on fibroblast expression or to the severe metabolic disturbance warrants further study [33, 34]. Due to the rarity of this condition, there are relatively few available patients to study and echocardiography is particularly limited by poor acoustic windows in ALMS patients. In our experience, CMR scanning takes longer and requires practice of breath holding techniques but is feasible in the great majority despite most adult ALMS patients having both severe hearing and visual impairment. Biomarkers would be of particular use in ALMS given the limitations of echocardiography and the limited availability of CMR but standard markers of cardiovascular stress, including NT pro-BNP were not useful. Future studies should consider the measurement of direct markers of collagen turnover and fibrosis, such as serum C-terminal propeptide of type I pro-collagen [35].

## Conclusion

Diffuse interstitial fibrosis is common in asymptomatic patients with ALMS and is associated with abnormalities of LV systolic and diastolic function. The presence of cardiac fibrosis may be actively modulated by the severity of metabolic dysfunction, with elevated triglycerides a potential marker or intermediary. Expansion of the ECV is an imaging biomarker of cardiovascular vulnerability that demands longitudinal study in ALMS.
